# IL-27 Enhances the Expression of TRAIL and TLR3 in Human Melanomas and Inhibits Their Tumor Growth in Cooperation with a TLR3 Agonist Poly(I:C) Partly in a TRAIL-Dependent Manner

**DOI:** 10.1371/journal.pone.0076159

**Published:** 2013-10-14

**Authors:** Yukino Chiba, Izuru Mizoguchi, Kana Mitobe, Kaname Higuchi, Hiroshi Nagai, Chikako Nishigori, Junichiro Mizuguchi, Takayuki Yoshimoto

**Affiliations:** 1 Department of Immunoregulation, Institute of Medical Science, Tokyo Medical University, Tokyo, Japan; 2 Department of Immunology, Tokyo Medical University, Tokyo, Japan; 3 Division of Dermatology, Department of Clinical Molecular Medicine, Kobe University Graduate School of Medicine, Kobe, Hyogo, Japan; Juntendo University School of Medicine, Japan

## Abstract

Interleukin (IL)-27 is a member of the IL-6/IL-12 cytokine family and possesses potent antitumor activity, which is mediated by multiple mechanisms. Toll-like receptor (TLR)3 is the critical sensor of the innate immune system that serves to identify viral double-stranded RNA. TLR3 is frequently expressed by various types of malignant cells, and recent studies reported that a synthetic TLR3 agonist, polyinosinic-polycytidylic acid [poly(I:C)], induces antitumor effects on malignant cells. In the present study, we have explored the effect of IL-27 on human melanomas and uncovered a previously unknown mechanism. We found that IL-27 inhibits in vitro tumor growth of human melanomas and greatly enhances the expression of TNF-related apoptosis inducing ligand (TRAIL) in a dose-dependent manner. Neutralizing antibody against TRAIL partly but significantly blocked the IL-27–mediated inhibition of tumor growth. In addition, IL-27 and poly(I:C) cooperatively augmented TRAIL expression and inhibited tumor growth. The cooperative effect could be ascribed to the augmented expression of TLR3, but not retinoic acid-inducible gene-I or anti-melanoma differentiation-associated gene 5, by IL-27. The inhibition of tumor growth by the combination was also significantly abrogated by anti-TRAIL neutralizing antibody. Moreover, IL-27 and poly(I:C) cooperatively suppressed in vivo tumor growth of human melanoma in immunodeficient mice. Taken together, these results suggest that IL-27 enhances the expression of TRAIL and TLR3 in human melanomas and inhibits their tumor growth in cooperation with poly(I:C), partly in a TRAIL-dependent manner. Thus, IL-27 and the combination of IL-27 and poly(I:C) may be attractive candidates for cancer immunotherapy.

## Introduction

Interleukin (IL)-27 is a heterodimeric cytokine belonging to the IL-6/IL-12 cytokine family and consists of an IL-12 p40-related protein, Epstein-Barr virus-induced gene 3, and an IL-12 p35-related protein, p28 [1]. T-cell cytokine receptor/WSX-1, which is homologous to the IL-12 receptor (R) β2 subunit, and gp130, a common receptor chain for the IL-6 cytokine family, constitute a functional signal-transducing receptor for IL-27 [1]. IL-27 activates signal transduction and activator of transcription (STAT)1 and STAT3 [2], [3] and induces both pro- and anti-inflammatory immune responses. IL-27 not only induces early helper T (Th)1 differentiation, but also suppresses Th2 and Th17 differentiation and pro-inflammatory cytokine production [4]. Moreover, IL-27 induces the differentiation into IL-10–producing regulatory T cells [5], [6].

We and other groups previously reported that IL-27 has a potent ability to induce tumor-specific antitumor and protective immunity through cytotoxic T lymphocyte (CTL) and natural killer (NK) cells in colon carcinoma colon 26 [7], [8] and neuroblastoma TBJ [9] lines. IL-27 was further demonstrated to exert antitumor activity against poorly immunogenic B16F10 melanoma, which is mediated through NK cells but not CTL [10], and also against NK cell–resistant head and neck squamous cell carcinoma SCCVII through NK cell–mediated antibody-dependent cellular cytotoxicity (ADCC) [11]. IL-27 was also shown to have potent anti-angiogenic activity by inducing anti-angiogenic chemokines, IFN-γ–inducing protein (IP-10, CXCL10) and monokine induced by IFN-γ (MIG, CXCL9), as does IFN-γ, but in an IFN-γ–independent manner [12]. In addition, we recently demonstrated that IL-27 has anti-proliferative activity and acts directly on melanomas through WSX-1/STAT1 signaling [13]. Thus, IL-27 exerts antitumor activities through multiple mechanisms including CTL, NK cells, ADCC, anti-angiogenic activity, and direct antiproliferative activity depending on the characteristics of individual tumors.

Moreover, it was recently demonstrated that IL-27 inhibits the growth of human tumors including melanoma, multiple myeloma, B-acute lymphoblastic leukemia, follicular lymphoma, diffuse large B-cell lymphoma, and acute myeloid leukemia [13]–[16]. IL-27 strongly inhibited tumor growth and in vivo tumorigenicity of multiple myeloma cells through suppression of angiogenesis [15]. IL-27 also severely hampered the leukemic spreading induced in nonobese diabetic/severe combined immunodeficiency (NOD/SCID)/IL-2Rγ^−/−^ mice by injection with B-acute lymphoblastic leukemia cells from pediatric patients [14]. Similarly, acute myeloid leukemia cells injected into NOD/SCID/IL-2Rγ^−/−^ mice gave rise to leukemia dissemination that was severely inhibited by IL-27 [16]. These antitumor effects were mainly attributed to significant reduction of angiogenic and spreading-related genes and also to up-regulation of angiostatic molecules [14]–[16].

Toll-like receptor (TLR) is a type of pattern recognition receptor and recognizes molecules that are broadly shared by pathogens but distinguishable from host molecules, collectively referred to as pathogen-associated molecular patterns. It plays a key role in the innate immune system, and once microbes have breached physical barriers such as the skin or intestinal tract mucosa, they are recognized by TLRs, which activate immune cell responses. TLR3 is a major effector of the immune response against viral pathogens, and it is involved in the early activation of NK and dendritic cells [17]. It is also expressed in a wide range of nonimmune cells in which it plays a crirical role in the induction of interferon response. Since TLR3 is frequently expressed by various types of malignant cells and can directly trigger tumor cell apoptosis, TLR3 is considered to be a promising target for therapeutic purpose [18]. One of the TLR3 agonists, polyinosinic-polycytidylic acid [poly(I:C)], has been demonstrated to induce potent antitumor activity against various tumors [19].

In the present study, we attempted to gain a better understanding of the molecular events governing the anti-proliferative effect of IL-27 on melanomas. We here show that IL-27 enhances the expression of TNF-related apoptosis inducing ligand (TRAIL) and TLR3 in human melanomas and therefore inhibits their tumor growth in cooperation with poly(I:C) partly in a TRAIL-dependent manner. This is the first report to show a role for TRAIL in IL-27–mediated inhibition of tumor growth as well as a cooperative effect between IL-27 and poly(I:C) on the inhibition of tumor growth through TLR3 up-regulation by IL-27.

## Materials and Methods

### Ethics statement

The animal study was approved by the institutional review board of Tokyo Medical University (S-22005, S-23044, S-24013 and S-25005).

### Cell culture and mice

Human melanoma cell lines, SK-MEL-13, -28, and -37 [20], were kindly provided by Drs. L. J. Old and G. Ritter (Ludwig Institute for Cancer Research, New York Branch, Memorial Sloan-Kettering Cancer Center, New York, NY) and Dr. T. Takahashi (Aichi Cancer Center, Nagoya, Japan), and cultured in Iscoves’s modified Dulbecco’s medium supplemented with 10% FBS. C57BL/6 mice and NOD/SCID mice were purchased from Sankyo Labo Service Corp. and Clea Japan, Inc., respectively.

### Reagents

Human and mouse recombinant IL-27 was prepared as a tagged single-chain fusion protein by flexibly linking EBI3 to p28 using HEK293-F cells (Life Technologies, Carlsbad, CA) as described previously [12]. Anti-phosphotyrosine (pY)-STAT1, anti-pY-STAT3, anti-TRAIL (C92B9), anti-retinoic acid-inducible gene-I (RIG-I, D14G6) and anti-melanoma differentiation-associated gene 5 (MDA5, D74E4) were purchased from Cell Signaling Technology (Danvers, MA). Anti-STAT1 and anti-STAT3 were from Santa Cruz Biotechnology (Dallas, TX). Anti-TRAIL and phycoerythrin (PE)-labeled anti-TRAIL (RIK-2) were from BioLegend (San Diego, CA). Anti-TLR3 (clone#512505) was from R&D Systems (Minneapolis, MN). Anti-β-actin and poly(I:C) were from Sigma-Aldrich (St. Louis, MO) and InvivoGen (San Diego, CA), respectively. Soluble TRAIL was purchased from Alexis Biochemicals (San Diego, CA).

### Western blotting

Cells were lysed in a lysis buffer containing protease inhibitors, and the resultant cell lysates were separated by SDS-PAGE under reducing conditions and transferred to polyvinylidene difluoride membrane (Millipore, Billerica, MA) as described previously [21]. The membrane was then blocked, probed with a primary antibody and then with an appropriate secondary antibody conjugated to HRP, and visualized with the enhanced chemiluminescence detection system (GE Healthcare, Uppsala, Sweden) according to the manufacturer’s instructions. Immunoreactive bands were detected with a ChemiDoc XRS (Bio-Rad, Hercules, CA), and the intensity of each band was quantified with the Image Lab (Bio-Rad) or Image J (NIH, Bethesda, MD) program.

### Proliferation assay

Cells (2×10^3^ to 8×10^3^ cells/200 µl/well) were stimulated with IL-27 and/or poly(I:C) for 48–96 h and pulsed with ^3^H-thymidine for the last 8–24 h. ^3^H-thymidine incorporation was counted using TopCount (PerkinElmer, Waltham, MA).

### Apoptosis assay

Apoptosis was assessed by flow cytometric analysis of cells stained with Annexin V-fluorescein isothiocyanate (FITC) and propidium iodide (PI) according to the manufacturer’s instruction (BD Biosciences, San Jose, CA).

### RT-PCR

Total RNA was extracted using a guanidine thiocyanate procedure, cDNA was prepared using oligo(dT) primer and SuperScript III RT (Life Technologies), and RT-PCR was performed using *Taq* DNA polymerase. Cycle conditions were 94°C for 40 s, 60°C for 20 s, and 72°C for 40 s. Primers used for gp130, WSX-1, and hypoxanthine phosphoribosyl transferase (HPRT) as an internal control were previously described [13], [22]. The following primers were also used: TRAIL sense primer, 5′-GTGCTGATCGTGATCTT-3′; TRAIL antisense primer, 5′-CCACTCCTTGATGATTCC-3′; TRAIL-R1 sense primer, 5′-CTGAGCAACGCAGACTCGCTGTCCAC-3′; TRAIL-R1 antisense primer, 5′-TCCAAGGACACGGCAGAGCCTGTGCCAT-3′; TRAIL-R2 sense primer, 5′-GCCTCATGGACAATGAGATAAAGGTGGCT-3′; TRAIL-R2 antisense primer, 5′-CCAAATCTCAAAGTACGCACAAACGG-3′; TRAIL-R3 sense primer, 5′-GAAGAATTTGGTGCCAATGCCACTG-3′; TRAIL-R3 antisense primer, 5′-CTCTTGGACTTGGCTGGGAGATGTG-3′; TRAIL-R4 sense primer, 5′-CTTTTCCGGCGGCGTTCATGTCCTTC-3′; TRAIL-R4 antisense primer, 5′-GTTTCTTCCAGGCTGCTTCCCTTTGTAG-3′; TLR3 sense primer, 5′-TTGCCTTGTATCTACTTTTGGGG-3′; TLR3 antisense primer, 5′-GCGGCTGGTAATCTTCTGAGTT-3′; RIG-I sense primer, 5′-TGTGGGCAATGTCATCAAAA-3′; RIG-I antisense primer, 5′-GAAGCACTTGCTACCTCTTGC-3′; MDA5 sense primer, 5′-GGCACCATGGGAAGTGATT-3′; MDA5 antisense primer, 5′-ATTTGGTAAGGCCTGAGCTG-3′; IFN regulatory factor (IRF)-1 sense primer, 5′-GAAGTCCAGCCGAGATGC-3′; IRF-1 antisense primer, 5′-CGGCACAACTTCCACTG-3′. Real-time quantitative PCR was carried out using SYBR Premix Ex Taq II (TAKARA, Otsu, Shiga, Japan) according to the manufacture’s instructions. Glyceraldehyde-3-phosphate (GAPDH) was used as house-keeping gene to normalize mRNA. Relative expression of real-time PCR products was determined by using the ΔΔCt method to compare target gene and GAPDH mRNA expression. Primers for TRAIL and GAPDH were purchased from TAKARA: TRAIL sense primer, 5′-CTTCACAGTGCTCCTGCAGTCTC-3′; TRAIL antisense primer, 5′-AAGGTAGACTTCAAGATGGCAGCAA-3′; GAPDH sense primer, 5′-GCACCGTCAAGGCTGAGAAC-3′; GAPDH antisense primer, 5′-TGGTGAAGACGCCAGTGGA-3′.

### Small interfering RNA (siRNA) transfection

Human TLR3 siRNA (ON-TARGETplus SMARTpool) and negative control siRNA (siGLO RISC-free) were purchased from Thermo Fisher Scientific (Lafayette, CO). Human RIG-I and MDA-5 siRNAs, whose sequences were reported previously [23], were synthesized by Sigma-Aldrich. Human melanoma cells of SK-MEL-37 cell line were plated in 12-well plates and transfected with these siRNAs using HiPerFect Transfection Reagent (Qiagen Inc., Venlo, Netherlands) or Lipofectamine RNAiMAX Transfection Reagent (Life Technologies) according to the manufacturers’ protocols.

### Human tumor xenograft model in immunodeficient mice

Human melanoma cells (5×10^6^ cells/mouse) of SK-MEL-37 cell line were injected subcutaneously (s.c.) into the right flank of NOD/SCID mice (n  =  4–5). The treatment was started 1 week postengraftment and consisted of weekly intravenous (i.v.) injections with PBS alone, mouse IL-27 (1 µg), poly(I:C) (30 µg), or mouse IL-27 (1 µg) plus poly(I:C) (30 µg) in 200 µl of PBS. Mouse and human IL-27 were previously demonstrated to almost equally inhibit human melanoma growth [13]. Tumor growth was monitored weekly with an electronic caliper and expressed as volume (mm^3^) by calculating using the following volume equation: 0.5(*ab* ^2^), where *a* is the long diameter and *b* is the short diameter.

### Statistical analysis

Statistical analysis was performed by Student’s t test. A *p* value of less than 0.05 was considered to indicate a statistically significant difference.

## Results

### IL-27 inhibits tumor growth of human melanomas

**Figure 1:**
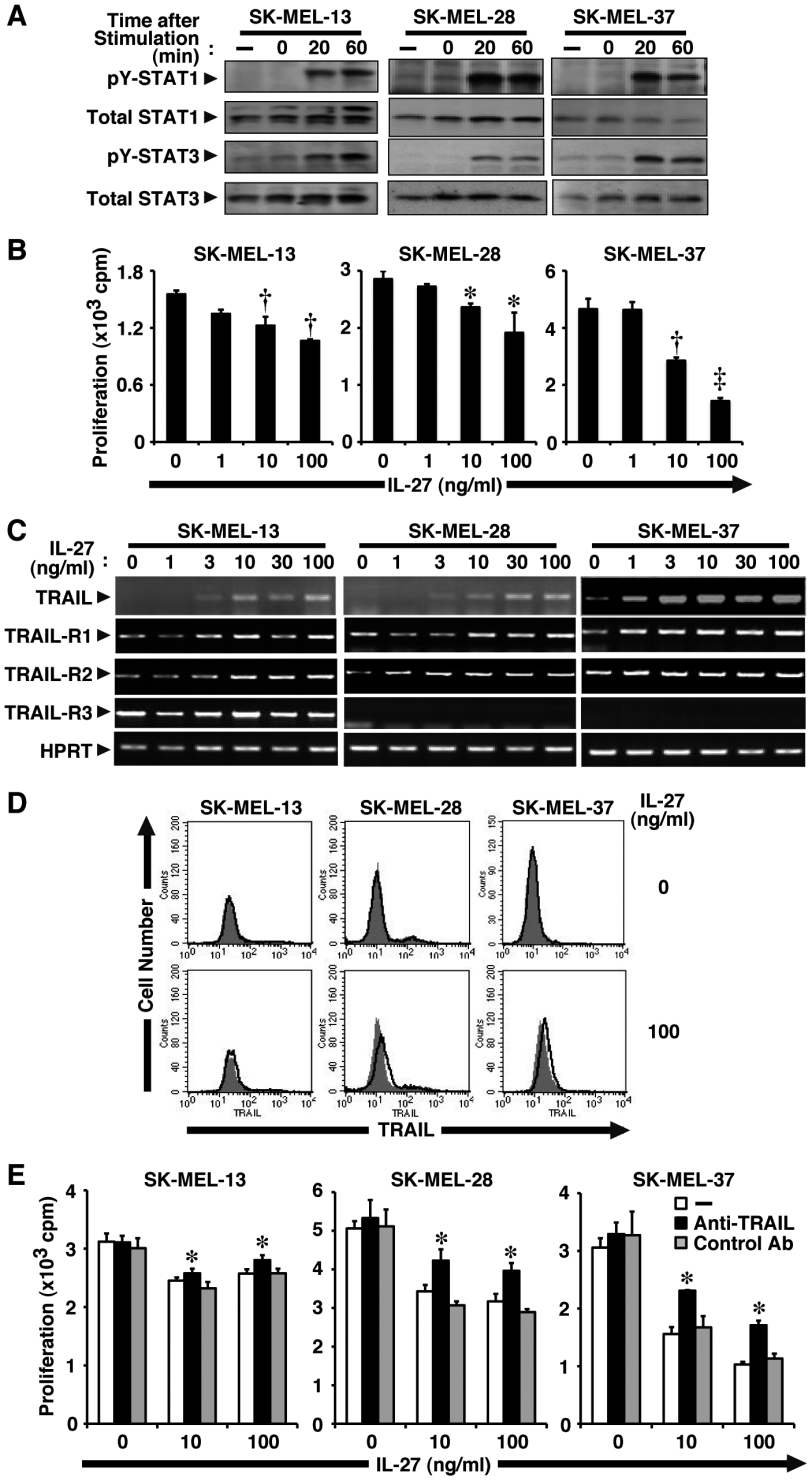
IL-27 induces TRAIL expression in human melanomas and inhibits their tumor growth partly in a TRAIL-dependent manner. (A) Human melanoma cell lines (SK-MEL-13, -28, and -37) were stimulated with IL-27 (10 ng/ml) for 0, 20, and 60 min. Cell lysate was then prepared and subjected to Western blotting with anti-pY-STAT1, anti-pY-STAT3, anti-total STAT1, and anti-total STAT3. Similar results were obtained in two independent experiments. (B) These melanoma cell lines were stimulated with increasing doses of IL-27 (0–100 ng/ml) for 72–96 h in triplicate and pulsed with ^3^H-thymidine for the last 24 h, and ^3^H-thymidine incorporation was measured. Data are shown as means ± SD. *, †, and ‡ indicate *p*<0.01, *p*<0.0001, and *p*<0.00001, respectively, compared with 0 ng/ml IL-27. Similar results were obtained in three independent experiments. (C) These melanoma cell lines were stimulated with increasing doses of IL-27 (0–100 ng/ml) for 24 h. Total RNA was then extracted and subjected to RT-PCR analysis. Similar results were obtained in more than three independent experiments. (D) These melanoma cell lines were stimulated with IL-27 (100 ng/ml) for 48 h, and then analyzed for cell surface expression of TRAIL by FACS using PE-labeled anti-TRAIL (solid line) and its control Ab (plain line with shading). Similar results were obtained in two independent experiments. (E) These melanoma cell lines were stimulated with IL-27 (10 and 100 ng/ml) in the presence of anti-TRAIL neutralizing Ab or its control Ab (10 µg/ml) for 72–96 h in triplicate and pulsed with ^3^H-thymidine for the last 24 h, and ^3^H-thymidine incorporation was measured. Data are shown as means ± SD. * indicates *p* < 0.05, compared with control Ab. Similar results were obtained in three independent experiments.

We previously demonstrated that mouse melanoma B16F10 cells are not responsive to IL-27 due to a lack of expression of one of the IL-27R subunits, WSX-1. However, B16F10 cells transfected with WSX-1-expression vector (B16F10-WSX-1) become as responsive to IL-27 as several human melanomas [13]. In three different human melanoma cell lines, SK-MEL-13, -28, and -37, IL-27 induced tyrosine phosphorylation of STAT1 and STAT3, indicating that these cells were responsive to IL-27 (Fig. 1A). Moreover, IL-27 dose-dependently inhibited their tumor growth (Fig. 1B). Among them, SK-MEL-37 seemed to be most susceptible to stimulation by IL-27.

### IL-27 induces TRAIL expression in human melanomas and inhibits their tumor growth partly in a TRAIL-dependent manner

We previously demonstrated that IL-27 inhibits the tumor growth of B16F10-WSX-1 cells through the WSX-1/STAT1 and IRF-1 pathway [13]. To gain a better understanding of the molecular events governing the anti-proliferative effect of IL-27 on melanomas, we first focused on TRAIL, which is an important immune effector molecule in the surveillance and elimination of developing tumors [24]. TRAIL is a promising antineoplastic agent because it induces apoptosis in cancer cells with only negligible effects on normal cells. In addition, several previous studies revealed that IFN-α induces anti-proliferative effects on cancer cells by stimulating them to produce TRAIL [25], [26]. First of all, we examined the sensitivity of three different cell lines of human melanoma to soluble TRAIL. Cell growth of all three cell lines was significantly inhibited in a dose-dependent manner, although the sensitivity appeared to vary among them (Fig. S1). Three human melanoma cell lines were then stimulated with increasing doses of IL-27, and total RNA was extracted 24 h later and subjected to RT-PCR analysis. The mRNA expression of TRAIL was greatly enhanced by IL-27 in a dose-dependent manner (Fig. 1C). Among the three melanoma cell lines, SK-MEL-37 cells expressed the highest TRAIL mRNA amount after stimulation by IL-27. At the protein level, IL-27 also enhanced the expression of TRAIL, which was detected by Western blot analysis (see the later section), but these melanomas only slightly expressed cell surface TRAIL in response to IL-27, which was detected by fluorescence-activated cell sorter (FACS) analysis (Fig. 1D).

We next examined whether or not these melanomas express TRAIL-Rs (TRAIL-R1–R4) [27] to transduce the signal by TRAIL. These melanomas expressed TRAIL-R1 and TRAIL-R2, and the expression levels appeared not to be affected by IL-27 (Fig. 1C). TRAIL-R3 and TRAIL-R4, known as decoy receptors-1 and -2, cannot induce apoptosis but inhibit TRAIL-induced apoptosis mediated by TRAIL-R1 and/or TRAIL-R2 when coexpressed [27]. TRAIL-R3 expression was detected in only SK-MEL-13 cells but not others, and its expression level was only minimally affected by IL-27 (Fig. 1C). Moreover, TRAIL-R4 expression was hardly detected among all these cell lines even after stimulation by IL-27 (data not shown). Thus, these melanomas would be susceptible to the stimulation by TRAIL, but the susceptibilities may vary among the melanoma lines.

To explore the role of enhanced expression of TRAIL on tumor growth, these melanomas were stimulated by IL-27 (10 and 100 ng/ml) in the presence of neutralizing antibody (Ab) against TRAIL or its control Ab. IL-27 inhibited tumor growth of melanomas, while anti-TRAIL Ab, but not control Ab, partly but significantly abrogated the inhibitory effect by IL-27 on tumor growth in all three melanoma cell lines (Fig. 1E).

These results suggest that IL-27 induces TRAIL expression at the mRNA level in human melanomas and inhibits tumor growth partly in TRAIL-dependent manner.

### IL-27 and TLR3 agonist poly(I:C) cooperatively inhibit tumor growth of human melanomas

**Figure 2:**
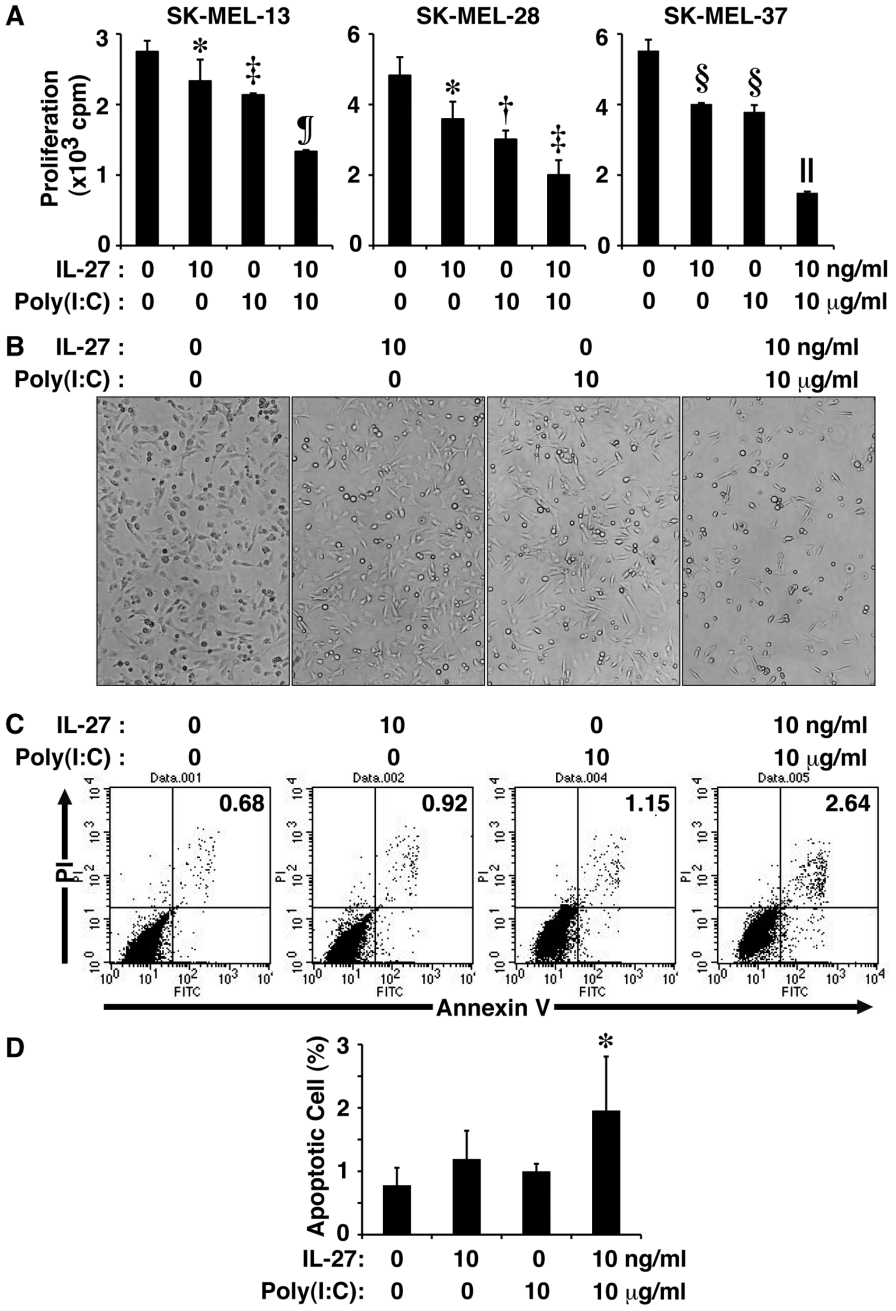
IL-27 and TLR3 agonist poly(I:C) cooperatively inhibits tumor growth of human melanomas. (A) Human melanoma cell lines (SK-MEL-13, -28 and -37) were stimulated with IL-27 (10 ng/ml) and/or poly(I:C) (10 µg/ml) for 72-96 h in triplicate and pulsed with ^3^H-thymidine for the last 24 h, and ^3^H-thymidine incorporation was measured. Data are shown as means ± SD. *, †, ‡, §, ¶, and || indicate *p*<0.05, *p*<0.01, *p*<0.001, *p*<0.00001, and *p*<0.000001, respectively, compared with 0 ng/ml IL-27 and 0 µg/ml poly(I:C). Similar results were obtained in thee independent experiments. (B) Morphology was evaluated using light microscopy. A representative result of SK-MEL-27 from among the three human melanoma cell lines is shown. Similar results were obtained in more than five independent experiments. (C) These melanoma cell lines were stimulated with IL-27 (10 ng/ml) and/or poly(I:C) (10 µg/ml) for 48 h. Then, apoptosis was assessed by FACS analysis of cells stained with Annexin V-FITC and PI. A representative result of SK-MEL-27 from among the three human melanoma cell lines is shown. Similar results were obtained in three independent experiments. (D) Percentage of each population was calculated. Data are shown as means ± SD of three independent experiments. * indicates *p* < 0.05, compared to no stimulation.

TLR3 is a critical sensor of the innate immune system that serves to identify viral double-stranded RNA [17]. Several recent studies suggested that TLR3 activation by its synthetic agonist poly(I:C) directly causes tumor cell apoptosis [18]. To enhance the anti-proliferative activity of IL-27 against melanomas, the combined effect of IL-27 and poly(I:C) on tumor growth was next explored. Three human melanoma cell lines were treated with IL-27 and/or poly(I:C) for 72–96 h and their proliferative responses were determined. Either IL-27 or poly(I:C) alone slightly reduced tumor growth but their combination more strongly inhibited tumor growth in the three melanoma cell lines (Fig. 2A). Examination of morphology of SK-MEL-37 melanoma cells also supported the enhanced inhibition of tumor growth by the combination (Fig. 2B). Similar phenomena were also observed in SK-MEL-13 and -28 melanomas (data not shown).

Annexin V binding to cell surface phosphatidylserine is one hallmark of apoptotic cell death, and PI, which stains DNA, is a marker for permeabilized, necrotic cells. The FACS analysis of SK-MEL-37 melanoma cells was then carried out after Annexin V-PI staining. The IL-27 and poly(I:C) combination slightly, but significantly, increased Annexin V-positive and PI-positive population containing apoptotic cells, but neither alone increased it under the experimental conditions (Fig. 2C and D). These results suggest that IL-27 mediates cytostatic activity against tumor cells rather than cytotoxic activity as reported [13]. A similar tendency was observed with the other melanomas, SK-MEL-13 and -28 (data not shown).

These results suggest that IL-27 and poly(I:C) cooperatively inhibit tumor growth of human melanomas.

### IL-27 enhances TLR3 expression in human melanomas, which could account for the cooperative effect between IL-27 and poly(I:C)

**Figure 3:**
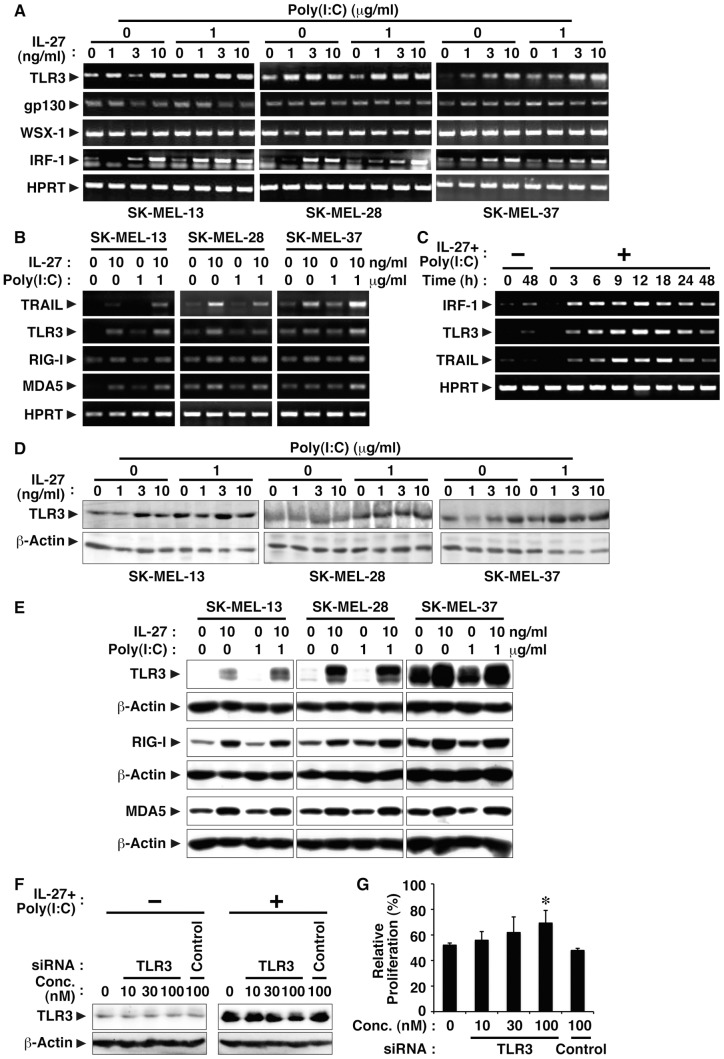
IL-27 enhances TLR3 expression in human melanomas, which could account for the cooperative effect between IL-27 and poly(I:C). (A-C) Human melanoma cell lines (SK-MEL-13, -28, and -37) were stimulated with IL-27 (1, 3, 10 ng/ml) and/or poly(I:C) (1 µg/ml) for 24 h or the indicated times. Total RNA was then extracted and subjected to RT-PCR analysis. Similar results were obtained in more than two independent experiments. (D and E) Cell lysate was also prepared after the stimulation for 48 h and subjected to Western blotting with antibodies against TLR3, RIG-I, MDA5 and β-actin. Similar results were obtained in more than two independent experiments. (F) SK-MEL-37 cells were transfected with siRNA specific to TLR3 or control siRNA for 24 h. These cells were then stimulated with IL-27 (10 ng/ml) and poly(I:C) (1 µg/ml) for a further 24 h, and total cell lysate was prepared and subjected to Western blot using anti-TLR3 and anti-β-actin. (G) The siRNA-transfected cells were also stimulated with IL-27 (10 ng/ml) and poly(I:C) (1 µg/ml) for a further 48 h and pulsed with ^3^H-thymidine for the last 8 h in triplicate. ^3^H-thymidine incorporation was measured, and relative proliferation (%) to that of respective unstimulated cells was calculated. Data are shown as means ± SD. * indicates *p* < 0.05 compared with no siRNA and control siRNA. Similar results were obtained in two independent experiments.

To clarify the molecular mechanism whereby IL-27 and poly(I:C) cooperatively inhibit tumor growth of melanomas, we next investigated the effect of IL-27 on the expression of TLR3, which is the receptor for poly(I:C), and also conversely the effect of poly(I:C) on the expression of IL-27R subunits, gp130 and WSX-1. Human melanomas were treated with increasing concentrations of IL-27 in the presence or absence of poly(I:C) for 24 h, and resultant cells were harvested. Total RNA and cell lysate were then prepared and subjected to RT-PCR and Western blot analyses, respectively. In all three cell lines, a slight amount of TLR3 expression was detected without any stimulation, while the expression was enhanced by IL-27 at both mRNA and protein levels (Fig. 3A and D). Moreover, the addition of poly(I:C) seemed to further augment it (Fig. 3A and D). Besides TLR3, RIG-I and MDA5 are also known to be important sensors for poly(I:C) in cytosols [28]. Therefore, we next examined the effect of IL-27 on the expression of these molecules. IL-27 enhanced their expression at both mRNA and protein levels (Fig. 3B and E). In contrast, these human melanomas express mRNA of the IL-27R subunits WSX-1 and gp130 even without any stimulation, and the addition of poly(I:C) hardly affected their expression (Fig. 3A).

Then, the involvement of up-regulated sensors for poly(I:C) in inhibiting SK-MEL-37 tumor growth by combining IL-27 and ploy(I:C) was investigated by knock-down of their expression using respective siRNAs. When up-regulated TLR3 expression was reduced by 58% with 100 nM of its siRNA, which was determined by the intensity of each band, suppressed tumor growth was significantly recovered (Fig. 3F and G). However, knock-down of RIG-I or MDA5 failed to affect the suppressed tumor growth (Fig. S2A-D).

We previously demonstrated that IL-27 induces IRF-1 expression, which is involved in the inhibition of tumor growth, in mouse melanoma B16F10 cells ectopically expressing WSX-1 [13]. In addition, IFN-γ and IFN-α were reported to enhance IRF-1-mediated TRAIL expression in human tumor cell lines [29], [30]. We then examined whether IL-27 induces IRF-1 expression in these human melanomas. Consistent with the previous results [13], [29], [31], IL-27 induced IRF-1 expression at the mRNA level in all three human melanomas (Fig. 3A). Moreover, time course analyses revealed that IRF-1 mRNA expression was rapidly induced in response to IL-27 and poly(I:C), which was followed by up-regulation of TLR3 and TRAIL (Fig. 3C).

These results suggest that IL-27 enhances the expression of TLR3 and TRAIL probably through IRF-1 in human melanomas, which could account for the cooperative effect between IL-27 and poly(I:C).

### IL-27 and poly(I:C) cooperatively induce TRAIL expression in human melanomas and inhibit tumor growth partly in a TRAIL-dependent manner

**Figure 4:**
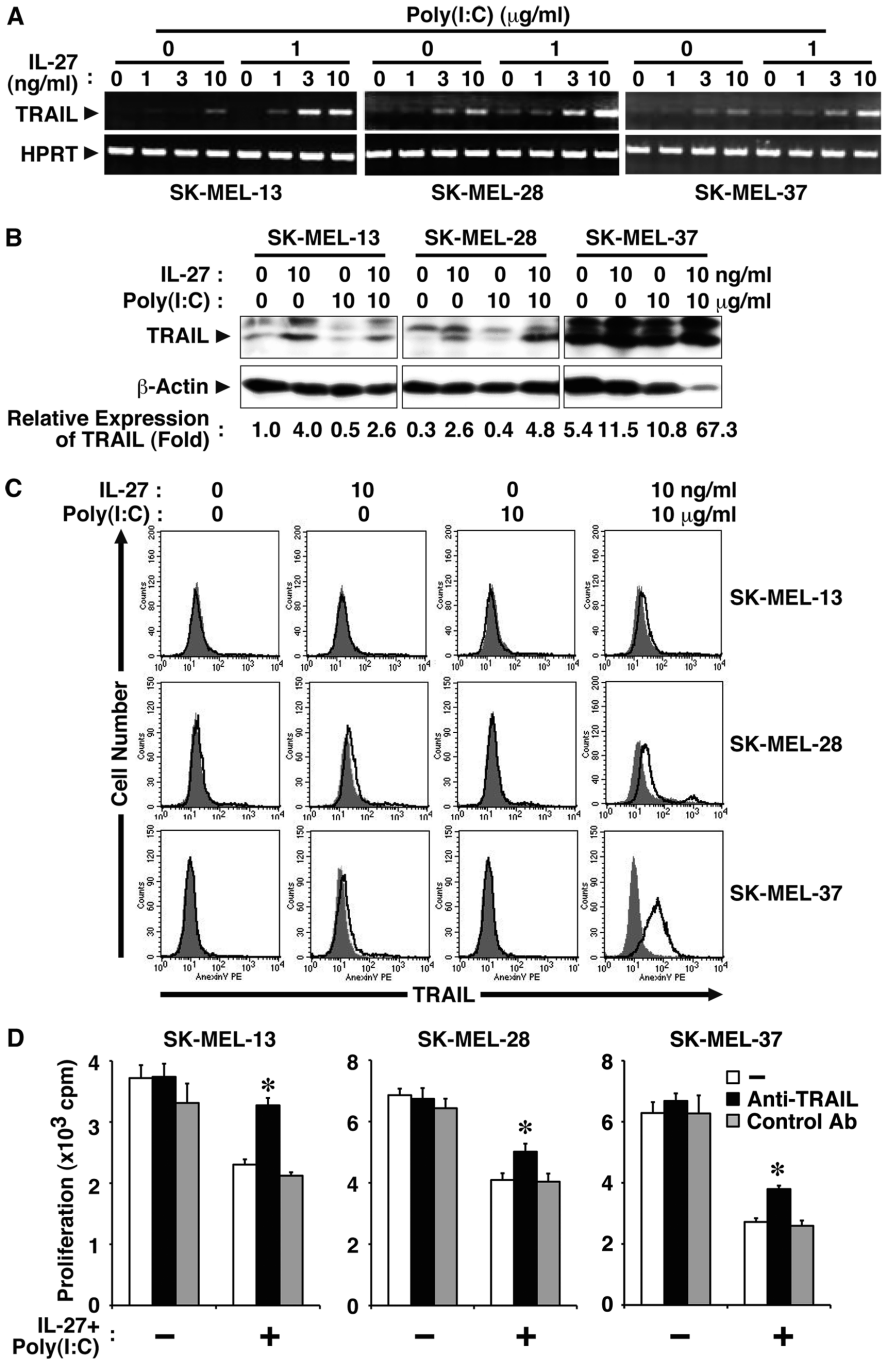
IL-27 and poly(I:C) cooperatively induce TRAIL expression in human melanomas and inhibit their tumor growth partly in a TRAIL-dependent manner. (A) Human melanoma cell lines (SK-MEL-13, -28, and -37) were stimulated with IL-27 (1, 3, 10 ng/ml) and/or poly(I:C) (0 or 1 µg/ml) for 24 h. Total RNA was then extracted and subjected to RT-PCR analysis. Similar results were obtained in three independent experiments. (B) These melanoma cell lines were stimulated with IL-27 (10 ng/ml) and/or poly(I:C) (10 µg/ml) for 48 h, and then total cell lysates were prepared and subjected to Western blot analysis using anti-TRAIL and anti-β-actin. Relative expression level of TRAIL was determined by the intensity of each band of TRAIL and β-actin. Similar results were obtained in two independent experiments. (C) These cells were also analyzed for cell surface expression of TRAIL by FACS using PE-labeled anti-TRAIL (solid line) and its control Ab (plain line with shading). Similar results were obtained in more than two independent experiments. (D) These melanoma cell lines were stimulated with IL-27 (10 ng/ml) and poly(I:C) (1 µg/ml) in the presence of anti-TRAIL neutralizing Ab or its control Ab (10 µg/ml) for 72–96 h in triplicate and pulsed with ^3^H-thymidine for the last 24 h, and ^3^H-thymidine incorporation was measured. Data are shown as means ± SD. * indicates *p* < 0.05, compared with control Ab. Similar results were obtained in three independent experiments.

To further demonstrate the cooperative effect between IL-27 and poly(I:C), we examined the effect of the combination on TRAIL expression in human melanomas. Three human melanoma cell lines were treated with increasing doses of IL-27 in the presence or absence of poly(I:C). Total RNA was extracted after 24 h and subjected to RT-PCR analysis. IL-27 enhanced the expression of TRAIL mRNA, and the presence of poly(I:C) further augmented it in all three melanomas (Fig. 4A). Then, total cell lysates were prepared after 48 h and subjected to Western blot analysis. Similar augmentation of TRAIL expression at protein levels was observed in these melanomas, but the expression level was highest in SK-MEL-37 and lowest in SK-MEL-13 (Fig. 4B). Cell surface expression of TRAIL was also analyzed by FACS. Synergistic induction of cell surface TRAIL expression by the combination of IL-27 and poly(I:C) was clearly observed in SK-MEL-28 and SK-MEL-37 cells 48 h after stimulation (Fig. 4C). However, only slight augmentation was detected in SK-MEL-13 cells 48 h but not 24 and 72 h (data not shown) after stimulation. Of note, regardless of the expression levels of TRAIL, the treatment with anti-TRAIL neutralizing Ab, but not control Ab, partly but significantly abrogated the inhibitory effect on tumor growth of combined IL-27 and poly(I:C) in all three melanoma cell lines (Fig. 4D).

These results suggest that IL-27 and poly(I:C) cooperatively induce TRAIL expression in human melanomas and significantly inhibit their tumor growth partly in a TRAIL-dependent manner. However, the TRAIL expression level induced by IL-27 and poly(I:C) seems to vary among three tumor cell lines; highest in SK-MEL-37 and lowest in SK-MEL-12 cells.

### IL-27 and poly(I:C) cooperatively inhibit in vivo tumor growth of human melanoma in immunodeficient mice

**Figure 5:**
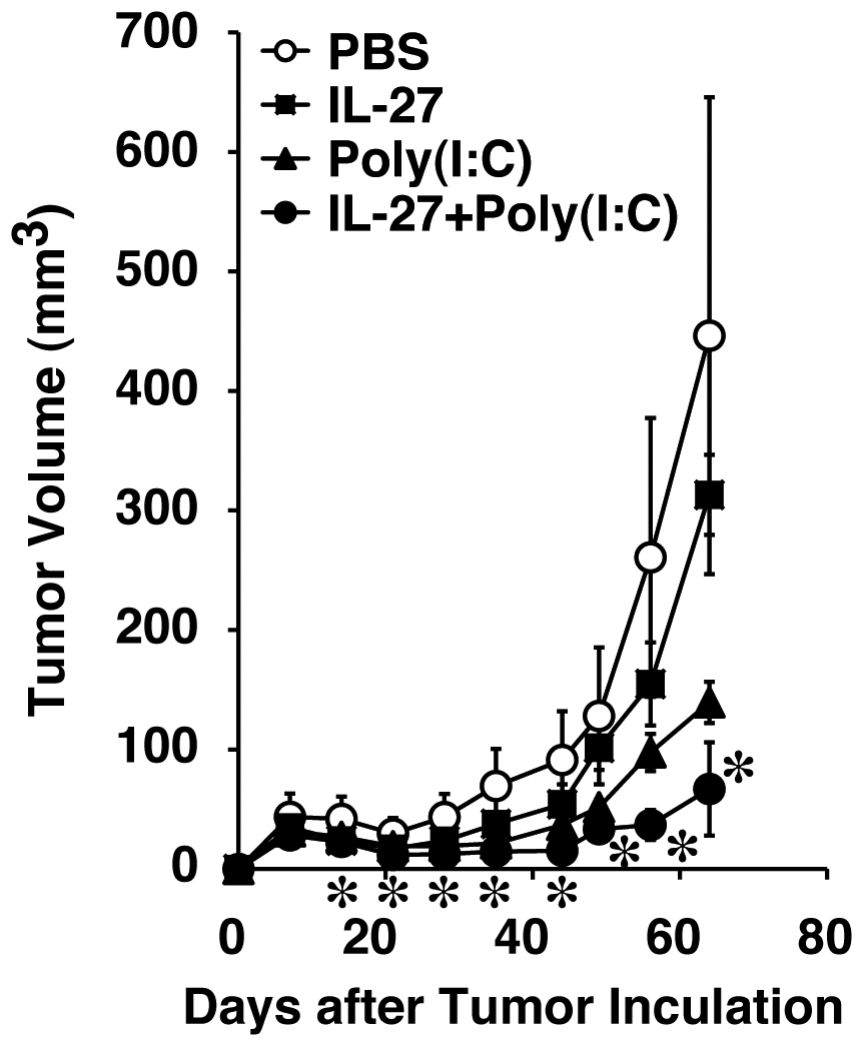
IL-27 and poly(I:C) cooperatively inhibit in vivo tumor growth of human melanoma in immunodeficient mice. Immunodeficient NOD/SCID mice were s.c. injected with human melanoma cells of the SK-MEL-37 cell line, and treated by weekly i.v. injections with PBS alone, IL-27 (1 µg), poly(I:C) (30 µg), or IL-27 (1 µg) plus poly(I:C) (30 µg) from 1 week postengraftment. Tumor growth was monitored weekly and expressed as volume (mm^3^). Data are shown as means ± SD. * indicates *p* < 0.05, compared with PBS. Similar results were obtained in two independent experiments.

Finally, in vivo antitumor effects of IL-27 or the combination of IL-27 and poly(I:C) were examined using a tumor xenograft mouse model in immunodeficient NOD/SCID mice. From among three human melanoma cell lines, SK-MEL-37 cells were used, because this cell line appears to be most susceptible to the treatment with IL-27 or combined IL-27 and poly(I:C) (Fig. 1B and 2A). NOD/SCID mice were s.c. injected with SK-MEL-37 cells, and treated by weekly i.v. injections with PBS, IL-27, poly(I:C), or IL-27 plus poly(I:C) from 1 week postengraftment. IL-27 alone only slightly suppressed tumor growth in vivo (Fig. 5). Poly(I:C) alone more greatly hampered in vivo tumor progression (Fig. 5), as reported previously [32]. Of note, the combination of IL-27 and poly(I:C) significantly inhibited tumor progression compared with PBS alone (Fig. 5). Then, the TRAIL expression at mRNA levels in the tumors was examined on day 63 after tumor injection. The TRAIL mRNA expression levels tended to increase by the IL-27 injections, although they were not significant (Fig. S3). Thus, these results suggest that IL-27 and poly(I:C) cooperatively inhibit in vivo tumor growth of human melanoma in immunodeficient mice.

## Discussion

**Figure 6:**
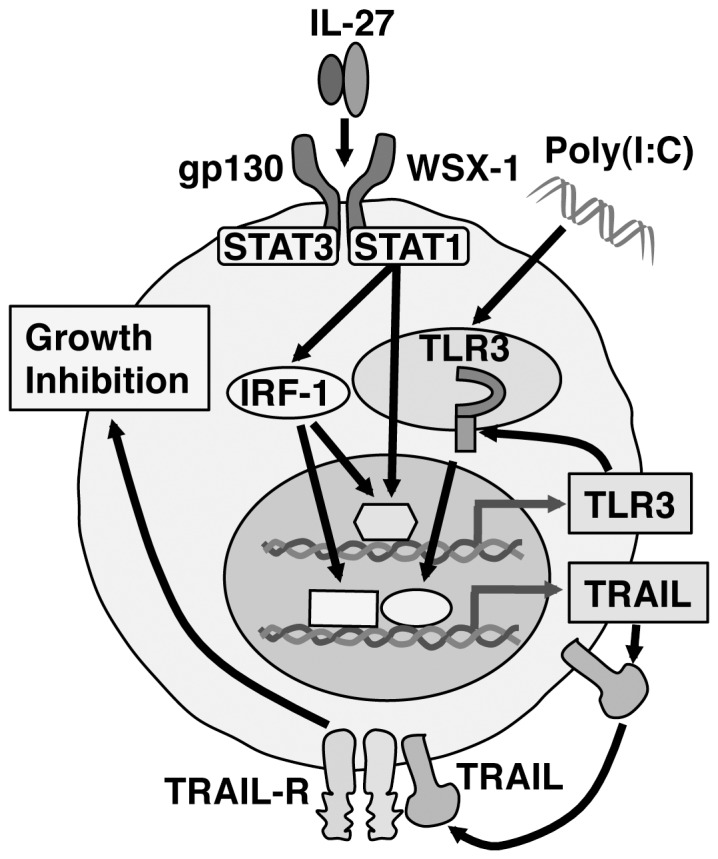
Hypothetical model of the pathway by which IL-27 and the combination of IL-27 and poly(I:C) induce TRAIL up-regulation and inhibits tumor growth in human melanomas. IL-27 induces IRF-1 expression through WSX-1/STAT1 signaling, resulting in up-regulation of TRAIL expression. IL-27 also augments TLR3 expression in IRF-1-dependent and -independent manner. Thus, due to the augmented TLR3 expression by IL-27, IL-27, and a synthetic TLR3 agonist, poly(I:C), cooperatively enhance TRAIL expression and greatly inhibit tumor growth partly in a TRAIL-dependent manner.

Since antitumor efficacy of IL-27 was first evaluated in 2004 [7], accumulating evidence has revealed that IL-27 has potent antitumor activity, which is mediated by multiple mechanisms including CD8^+^ T cells [7]–[9], [33], NK cells [10], [34], ADCC [11], anti-angiogenesis [12], [14]–[16], [35], [36], direct suppression of tumor growth [13], and inhibition of cyclooxygenase-2 expression [37], depending on the characteristics of individual tumors. In the present study, we further explored the molecular mechanism whereby IL-27 induces direct suppression of tumor growth of human melanomas. We elucidated that IL-27 augments the expression of TRAIL and TLR3 together with RIG-I and MDA5 in human melanomas, and that IL-27 and a TLR3 agonist, poly(I:C), cooperatively enhance TRAIL expression and inhibit their tumor growth partly in a TRAIL-dependent manner as illustrated in Fig. 6. The cooperative effect could be ascribed to the enhanced expression of TLR3, but not RIG-I or MDA5, by IL-27 (Fig. 3A, B, D-G and Fig. S2A-D), which is consistent with facts that RIG-I and MDA5 sense transfected poly(I:C), but not naked poly(I:C), in non-phagocytic cells [38], [39]. Since IL-27 augmented only marginal apoptosis even in collaboration with poly(I:C) (Fig. 2C and D), IL-27 is considered to mediate cytostatic activity rather than cytotoxic activity as reported before [13]. Furthermore, the combination of IL-27 and poly(I:C) significantly suppressed in vivo tumor progression in the human melanoma xenograft model using immunodeficient NOD/SCID mice (Fig. 5). When a higher dose (100 µg) of poly(I:C) was injected, tumor progression was more markedly inhibited regardless of the combination with IL-27 (data not shown) as reported previously [32], [40]. However, we noticed that 3 out of 5 mice died even without any tumor after the experiment was completed. In contrast, intriguingly, no mice died with treatment based on combined IL-27 and poly(I:C) (data not shown). Although further studies are necessary, the combination treatment may be beneficial in reducing the potential toxicity arising from a higher dose of poly(I:C) [41].

Among three human melanoma cell lines used in this study, SK-MEL-13 showed slightly less susceptibility to the IL-27 and poly(I:C)–mediated TRAIL-dependent growth inhibition and SK-MEL-37 showed more susceptibility to it (Fig. 1B and Fig. 2A). This tendency is consistent with the enhanced expression levels of TRAIL (Fig. 4B and C) and also TLR3 (Fig. 3D and E) in response to IL-27 and poly(I:C), although the expression levels of the receptors gp130, WSX-1, RIG-I and MDA5 appeared to be comparable (Fig. 3A, B and E). This tendency might be also affected by the constitutive expression of the decoy receptor for TRAIL, TRAIL-R3 (Fig. 1C), which inhibits TRAIL signaling [27], or by the different sensitivity to soluble TRAIL (Fig. S1). Of note, anti-TRAIL neutralizing Ab partly but significantly abrogated the IL-27 and poly(I:C)–mediated growth inhibition in all these cell lines (Fig. 1E and 4D). This partial abrogation might be due to the insufficient ability of the Ab to neutralize the TRAIL activity, or alternatively, there could be TRAIL-independent mechanisms as recently reported in the mechanism for IFN-induced apoptosis [42]. DNase II-deficient embryos die in utero due to severe anemia caused by IFN-β produced in the macrophages carrying undigested DNA. Although a high level of TRAIL mRNA was found in the fetal liver, a null mutation in TRAIL failed to rescue the lethal anemia, indicating that TRAIL is not necessary for inducing the apoptosis of erythroid cells in DNase II-deficient embryos [42]. Further studies are necessary to clarify the molecular mechanisms underlying IL-27 and poly(I:C)–mediated inhibition of tumor growth in TRAIL-dependent and -independent manners.

The IFNs, especially type I IFNs such as IFN-α and -β, are well-investigated cancer therapeutic agents [43]. IFN-α is used for the treatment of renal cell carcinoma, leukemia, and malignant melanoma. Several previous studies reported that IFN-α and IFN-γ induces anti-proliferative effects on a variety of cancer cells by stimulating them to produce TRAIL [25], [26]. IFN-α and IFN-γ enhance TRAIL expression via a STAT1– and IRF-1–dependent mechanism in cancer cells [29], [30]. We previously demonstrated that IL-27 induces IRF-1 expression in mouse B16F10 melanoma cells ectopically expressing wild-type WSX-1, but not in those expressing WSX-1 mutated in the tyrosine residue critical for STAT1 binding [13]. Moreover, the induction of IRF-1 was revealed to contribute to the IL-27–mediated inhibition of tumor growth of B16F10 melanoma [13]. Taken together with the present results, it is highly conceivable that IL-27–induced up-regulation of TRAIL in human melanomas could be also mediated by STAT1/IRF-1 signaling (Fig. 6).

TLR agonists are currently being studied in many clinical immunotherapy trials as vaccine adjuvants. TLR3, a member receptor of double-stranded RNAs, is a major effector of the immune response against viral pathogens at the cellular and systemic level [44]. It is involved in early activation of NK and dendritic cells and expressed in a wide range of nonimmune cells in which it plays a key role in the induction of the IFN response [45]. TLR3 is also frequently expressed by various types of malignant cells, and several studies reported that the synthetic TLR3 agonist, poly(I:C), induces proliferation blockade and apoptosis in malignant cells in vitro and in vivo [18], [19], [32], [40], [46]. Moreover, it was reported that IFN-α up-regulates the expression of TLR3 in lung carcinoma A549 cells and human umbilical vein endothelial cells [47]. Although IFN-α by itself did not induce massive apoptosis, it increased TLR3 mRNA and enhanced the anti-proliferative effect of poly(I:C). Inhibiting the JAK/STAT pathway diminished the induction of TLR3 by IFN-α, which highlights the importance of JAK/STAT signaling on IFN-α–induced TLR3 transcription. It was previously noted that the TLR3 promoter contains an IFN-stimulated response element and a STAT binding site, which were suggested to be involved in regulating the response of TLR3 to IFN-α [48]. In addition, it was previously reported that IFN-β induces TLR3 up-regulation through IFN-α receptor 1, STAT1, and in part IRF-1, but not Tyk2, in murine macrophages [49]. Similar to IFN-α and IFN-β, IL-27 activates STAT1 and induces IFN-inducible genes including antiviral genes, resulting in inhibition of HIV replication in CD4^+^ T cells and macrophages [50]. These reports indicate similarity between IFN-α/β and IL-27 in the signaling events and biological functions as previously suggested [51], which could account for the IL-27–induced up-regulation of TLR3 and TRAIL in human melanomas.

TLR4 is important in mediating inflammatory cytokine production in response to bacterial infection, and LPS is the main ligand binding to TLR4 to induce inflammation. Interestingly, although engagement of TLR3 expressed in human breast tumor cells induces massive apoptosis [18], triggering TLR4 in murine colon cancer MC26 cell line leads to immune evasion mediated by the inhibition of T- and NK-cell activities [52]. Similar to the present results, it has very recently been reported that IL-27 enhances LPS-induced proinflammatory cytokine production through up-regulation of TLR4 expression in human monocytes [53]. However, this up-regulation was mediated via JAK2, STAT3 and NF-κB, but not via STAT1. Thus, requirements of STAT1 and STAT3 in the signaling for up-regulation of distinct TLRs by IL-27 appear to differ between TLR3 and TLR4. Further studies are necessary to precisely elucidate the molecular mechanism whereby IL-27 induces up-regulation of TLR3 and TLR4.

Taken together, the present results suggest that IL-27 exerts anti-proliferative activity against human melanomas by mechanisms similar to those of IFN-α in a STAT1/IRF-1–dependent manner and partly in a TRAIL-dependent manner. Moreover, IL-27 increases TLR3 expression and therefore the combination of IL-27 and poly(I:C) cooperatively enhances TRAIL expression and inhibits tumor growth. IL-12 is one of the cytokines with strong antitumor activity. The induction of the antitumor activity and establishment of protective immunity by IL-12 are highly dependent on production of IFN-γ by NK and T cells [54]. However, this high IFN-γ production also causes systemic toxicities, which leads to limitation of the IL-12 therapy in clinical trials [55]. In this regards, IL-27 has much less toxicities compared with IL-12, probably due to much lower ability of IL-27 to produce IFN-γ by NK and T cells [7], [10]. Therefore, IL-27 and the combination of IL-27 and poly(I:C) may be attractive candidates as antitumor agents applicable to cancer immunotherapy.

## Supporting Information

Figure S1 **Human melanoma cell lines SK-MEL-13, 28 and 37 are sensitive to soluble TRAIL.** Three melanoma cell lines were stimulated with increasing doses of soluble TRAIL (0–100 ng/ml) for 48 h in triplicate and pulsed with ^3^H-thymidine for the last 24 h, and ^3^H-thymidine incorporation was measured. Data are shown as means ± SD. *, *p*<0.01, compared with 0 ng/ml soluble TRAIL. Similar results were obtained in two independent experiments.
(TIF)

Figure S2 **Knock-down of RIG-I or MDA5 hardly affected the tumor growth suppressed by combining IL-27 and ploy(I:C).** SK-MEL-37 cells were transfected with siRNAs specific to RIG-I (A and B), MDA5 (C and D) or control siRNA for 24 h. These cells were then stimulated with IL-27 (10 ng/ml) and poly(I:C) (1 µg/ml) for a further 24 h, and total cell lysate was prepared and subjected to Western blot using anti-RIG-I (A), anti-MDA5 (C) and anti-β-actin. The siRNA-transfected cells were also stimulated with IL-27 (10 ng/ml) and poly(I:C) (1 µg/ml) for a further 48 h and pulsed with ^3^H-thymidine for the last 24 h in triplicate. ^3^H-thymidine incorporation was measured, and relative proliferation (%) to that of respective unstimulated cells was calculated (B and D). Data are shown as means ± SD. * indicates *p* < 0.05 compared with no siRNA and control siRNA. Similar results were obtained in two independent experiments.
(TIF)

Figure S3 **The TRAIL mRNA expression levels in the tumors tended to increase by the IL-27 injections into tumor-bearing mice, although they were not significant.** Immunodeficient NOD/SCID mice were s.c. injected with human melanoma cells of the SK-MEL-37 cell line, and treated by weekly i.v. injections with PBS alone, IL-27 (1 µg), poly(I:C) (30 µg), or IL-27 (1 µg) plus poly(I:C) (30 µg) from 1 week postengraftment. Tumor growth was monitored weekly, and on day 63 after tumor injection TRAIL expression levels in the tumor sites were compared by using real-time quantitative PCR.
(TIF)
