# Supplementary figures and images for: IL-27 Enhances the Expression of TRAIL and TLR3 in Human Melanomas and Inhibits Their Tumor Growth in Cooperation with a TLR3 Agonist Poly(I:C) Partly in a TRAIL-Dependent Manner
Source: PLoS One. 2013 Oct 14;8(10):e76159. doi: 10.1371/journal.pone.0076159 (PMC3796519; doi:10.1371/journal.pone.0076159)

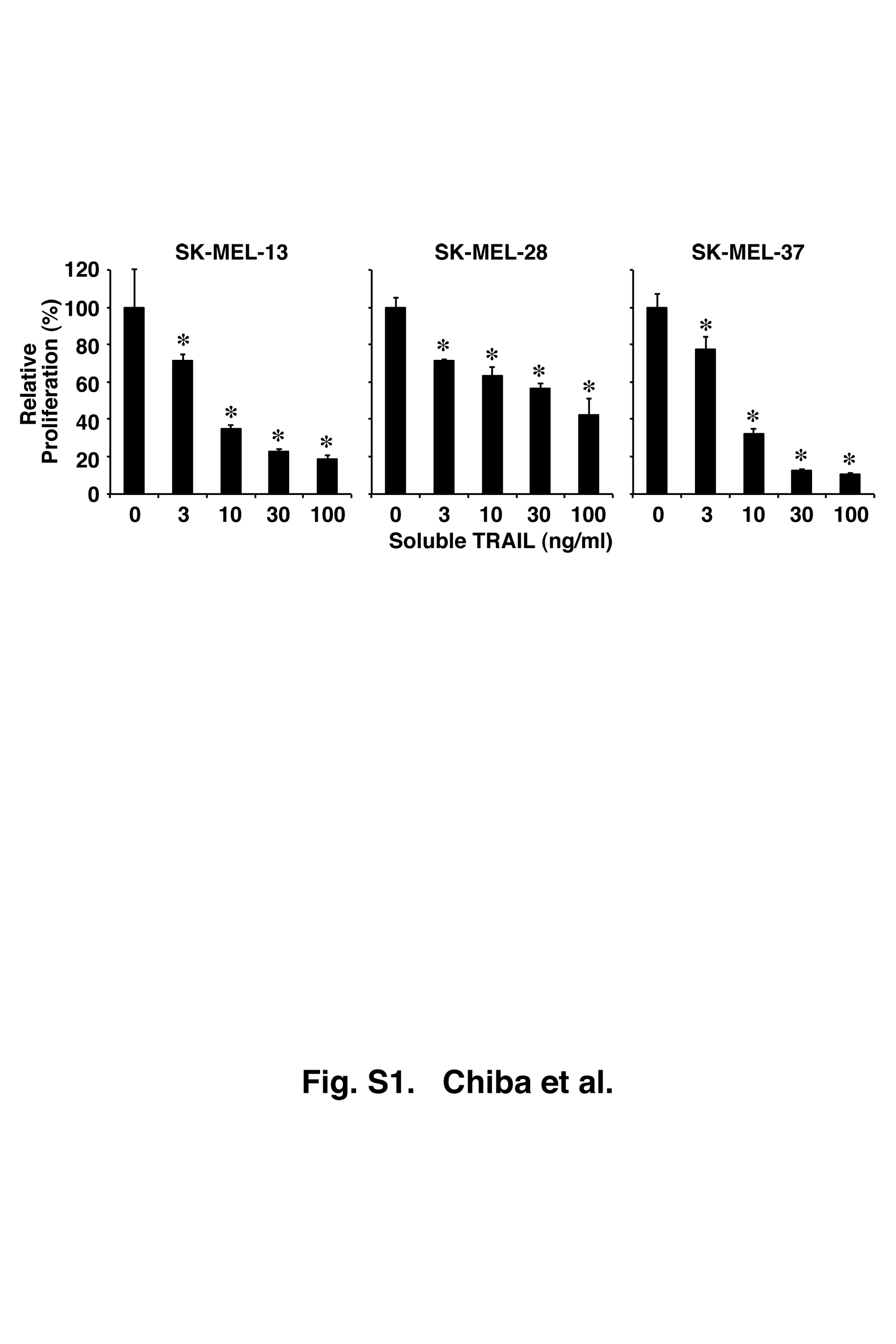

Supplement: Figure S1 — Human melanoma cell lines SK-MEL-13, 28 and 37 are sensitive to soluble TRAIL. Three melanoma cell lines were stimulated with increasing doses of soluble TRAIL (0–100 ng/ml) for 48 h in triplicate and pulsed with 3H-thymidine for the last 24 h, and 3H-thymidine incorporation was measured. Data are shown as means ± SD. *, p<0.01, compared with 0 ng/ml soluble TRAIL. Similar results were obtained in two independent experiments. (TIF) [file pone.0076159.s001.tif]

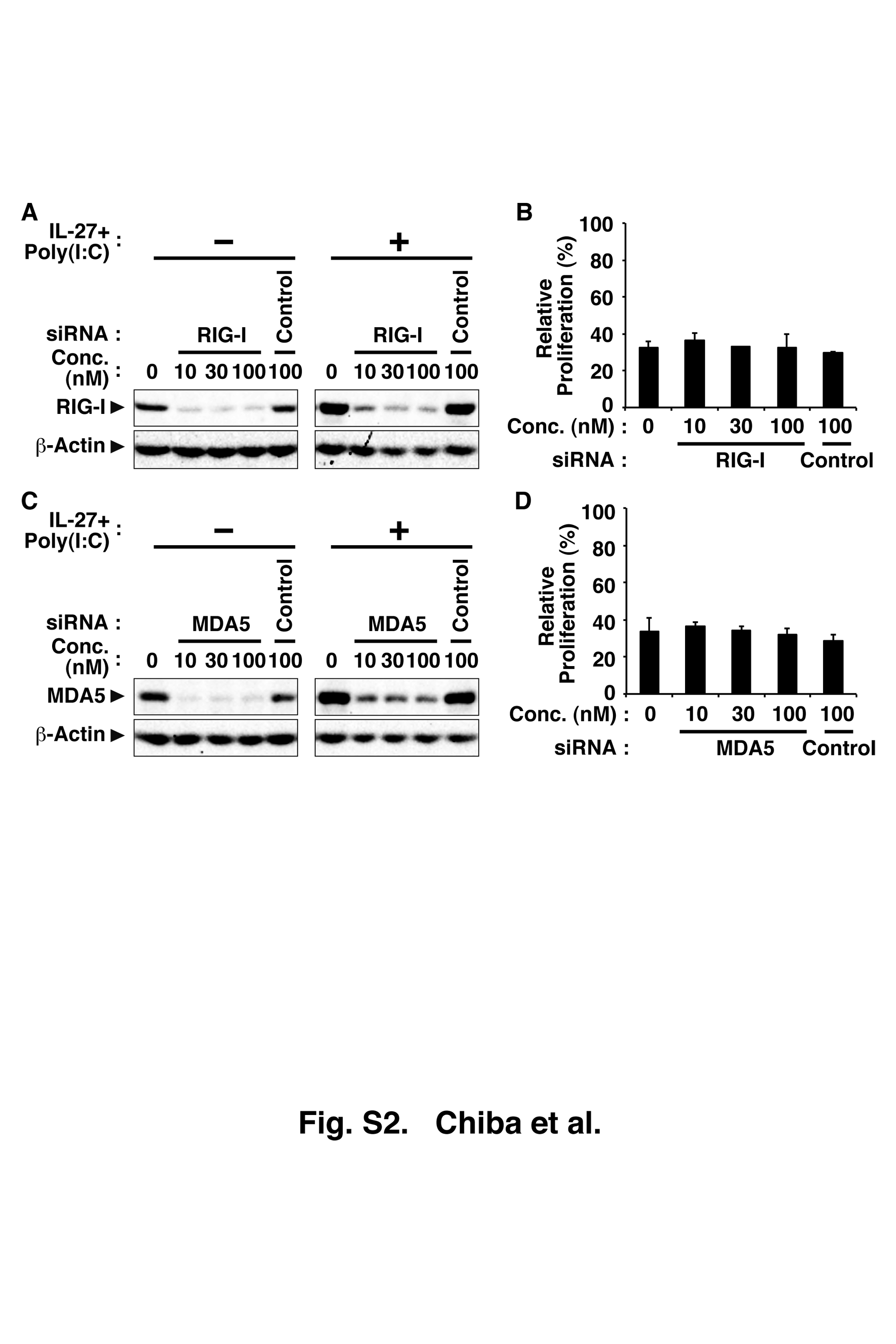

Supplement: Figure S2 — Knock-down of RIG-I or MDA5 hardly affected the tumor growth suppressed by combining IL-27 and ploy(I:C). SK-MEL-37 cells were transfected with siRNAs specific to RIG-I (A and B), MDA5 (C and D) or control siRNA for 24 h. These cells were then stimulated with IL-27 (10 ng/ml) and poly(I:C) (1 µg/ml) for a further 24 h, and total cell lysate was prepared and subjected to Western blot using anti-RIG-I (A), anti-MDA5 (C) and anti-β-actin. The siRNA-transfected cells were also stimulated with IL-27 (10 ng/ml) and poly(I:C) (1 µg/ml) for a further 48 h and pulsed with 3H-thymidine for the last 24 h in triplicate. 3H-thymidine incorporation was measured, and relative proliferation (%) to that of respective unstimulated cells was calculated (B and D). Data are shown as means ± SD. * indicates p < 0.05 compared with no siRNA and control siRNA. Similar results were obtained in two independent experiments. (TIF) [file pone.0076159.s002.tif]

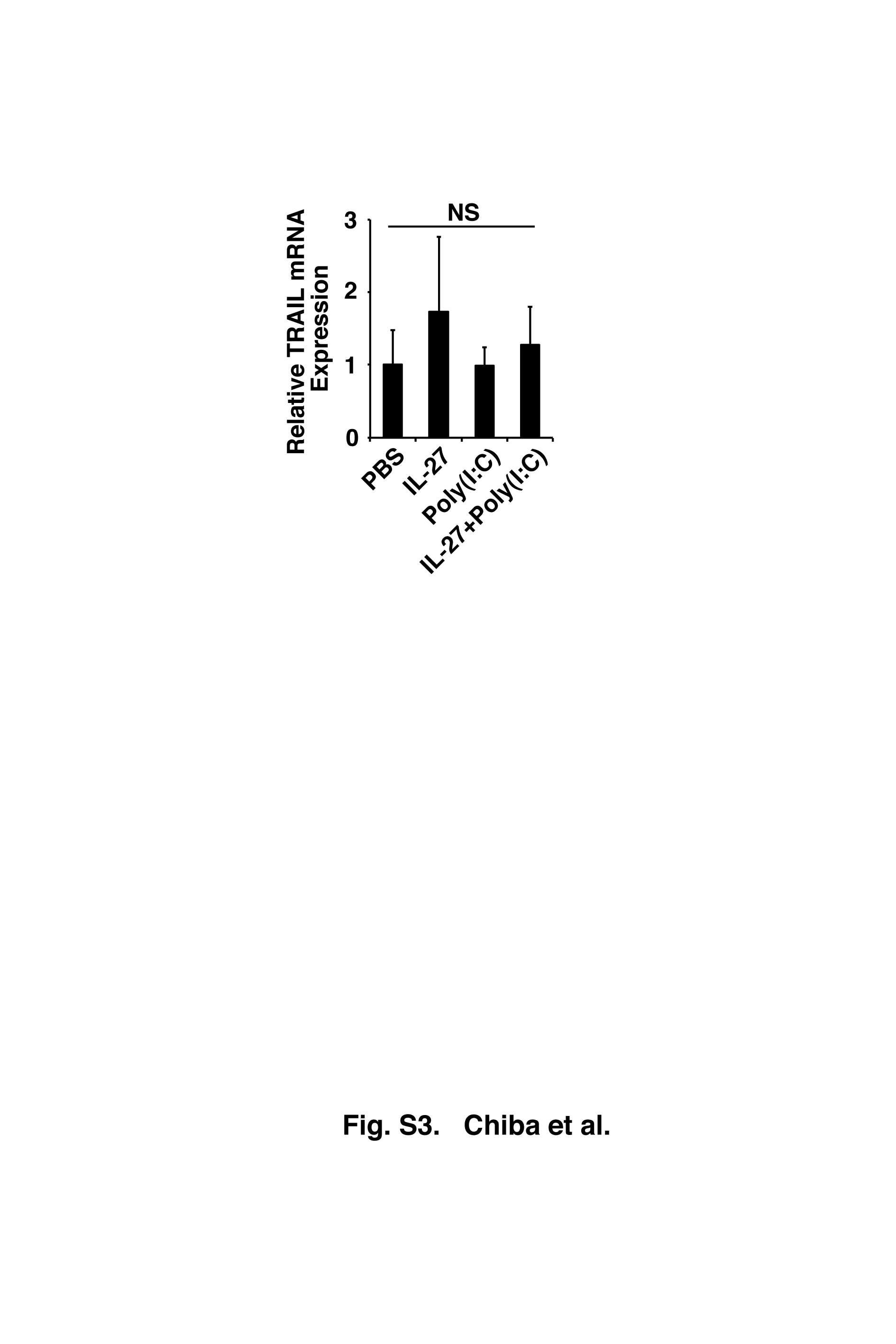

Supplement: Figure S3 — The TRAIL mRNA expression levels in the tumors tended to increase by the IL-27 injections into tumor-bearing mice, although they were not significant. Immunodeficient NOD/SCID mice were s.c. injected with human melanoma cells of the SK-MEL-37 cell line, and treated by weekly i.v. injections with PBS alone, IL-27 (1 µg), poly(I:C) (30 µg), or IL-27 (1 µg) plus poly(I:C) (30 µg) from 1 week postengraftment. Tumor growth was monitored weekly, and on day 63 after tumor injection TRAIL expression levels in the tumor sites were compared by using real-time quantitative PCR. (TIF) [file pone.0076159.s003.tif]
